# A Nuanced Look at Women in STEM Fields at Two-Year Colleges: Factors That Shape Female Students' Transfer Intent

**DOI:** 10.3389/fpsyg.2017.00146

**Published:** 2017-02-06

**Authors:** Xueli Wang, Hsun-yu Chan, Sara Jimenez Soffa, Brett Ranon Nachman

**Affiliations:** ^1^Educational Leadership and Policy Analysis, University of Wisconsin-MadisonMadison, WI, USA; ^2^Department of Educational Psychology, Texas A&M UniversityCommerce, TX, USA; ^3^Educational Leadership, Edgewood CollegeMadison, WI, USA

**Keywords:** two-year college, community college, women in STEM, math self-efficacy, science self-efficacy, transfer-oriented interaction

## Abstract

In this study, we explored the relationship between the intent to transfer upward and a set of motivational, contextual, and socio-demographic background factors among 696 female students beginning in science, technology, engineering, and mathematics (STEM) programs or courses at two-year colleges in a Midwestern state. Drawing upon survey data and administrative records, our multinomial logistic regression analysis revealed that students' math and science self-efficacy beliefs, as well as transfer-oriented interaction, were significant and positive predictors for their intent to transfer into STEM fields as opposed to having no intent to transfer. In addition, the association between transfer intent and these key motivational and contextual factors was moderated by students' racial/ethnic backgrounds, marital status, and childcare obligations. For example, despite the positive relationship between transfer-oriented interaction and the intention to transfer into STEM fields, Black women were less likely to have intent to transfer into STEM fields than White students until Black students reported a moderate level of transfer-oriented interaction. Conversely, Hispanic students were more likely to report intent to transfer into STEM fields than their White peers, even when Hispanic students reported a relatively low level of engagement in transfer-oriented interaction. These and other reported findings bear important and nuanced implications as policymakers, educators, and researchers continue to discover ways to better support women's educational pathways and success in STEM fields at and through two-year colleges.

## Background of the study

An increasing national demand to employ baccalaureate graduates in science, technology, engineering, and mathematics (STEM) careers has called for robust research on students' decisions and pathways to pursue these postsecondary fields of study. Carnevale et al. (2010) indicated that, by 2018, ~42% of STEM employment opportunities will require workers to possess a baccalaureate degree. A more pressing challenge within this endeavor is to resolve the severe gender gaps in the participation and completion rates of baccalaureate STEM programs (Ma, 2011). Historically, these programs are dominated by male students, with women seriously underrepresented (Riegle-Crumb and King, 2010). As such, supporting female students' pathways and success in STEM disciplines at the baccalaureate level is critical to ameliorating the human resource gap in these fields (Espinosa, 2011).

To close the noted gender gap, two-year colleges play an important role by serving as a potential pathway to four-year institutions for women pursuing baccalaureate STEM degrees (Christian, 2000; Boswell, 2004; Cohen et al., 2014). Historically, community and technical colleges are celebrated for their accessibility, affordability, and open admissions (Johnson et al., 2000; Cohen et al., 2014; Chavez, 2015). In recent years, emerging partnerships between four-year institutions and two-year colleges in supporting underrepresented students to pursue baccalaureate and/or graduate degrees in STEM fields provide realistic pathways for students who may not have otherwise considered transfer (Hirst et al., 2014). In particular, two-year colleges are increasingly recognized as entry points for women who plan to pursue degrees in STEM fields (Jackson et al., 2013). Compared to four-year colleges and universities, two-year colleges serve proportionately more women (Horn et al., 2006). Specifically, women make up 58% of students enrolled in two-year colleges (Phillippe and Patton, 2000; Bryant, 2001), with recent statistics showing over four million women enrolled at public two-year institutions (St. Rose and Hill, 2013). Female students' graduation rates also outshine their male counterparts, as women accounted for 62% of those who earned associate degrees during the 2009–2010 academic year (Cohen et al., 2014). Moreover, many women enrolled at two-year colleges also come from historically underserved populations, such as first-generation, racial/ethnic minority, low-income, and part-time students (Horn et al., 2006; Snyder and Dillow, 2012). As such, two-year colleges are uniquely positioned to expand both the number and diversity of women holding a STEM baccalaureate through the upward transfer function.

Despite this potential of two-year colleges for broadening women's participation in baccalaureate STEM programs, many baccalaureate-aspiring women starting in STEM fields at two-year colleges meet unexpected roadblocks to their pursuit of a four-year STEM degree, and consequently drop their intent to transfer (St. Rose and Hill, 2013). Empirical research is extremely limited to shed a holistic light on factors that influence these women's intent to transfer. In addition, research that examines the experiences of women in STEM fields is narrow in scope. Few studies have delved into how women's other identities, such as race/ethnicity, marital, and parental status, as well as first-generation status, shape their educational intent along the STEM pathways. These other characteristics and life responsibilities are particularly relevant to women at two-year colleges, considering the vast amount of diversity among this student population. Women outnumber men across all racial/ethnic backgrounds at two-year colleges, and 30% of women at two-year colleges identify as African American or Latina (Morganson et al., 2010; St. Rose and Hill, 2013). Adult women often select two-year colleges as their entry or reentry into college because these institutions are more conducive to balancing work, families, and school responsibilities (Johnson et al., 2000). Furthermore, low-income women and women with dependent children often choose two-year colleges (Costello, 2012). These background characteristics and factors may very well-intersect with other learning and motivational factors to collectively influence two-year college women's educational intent and outcomes in STEM. Yet, existing literature offers little insight into these nuances.

Aiming to address these important gaps in the literature, our study explores factors associated with the intent to transfer upward among female students beginning in STEM programs or courses at two-year colleges. In addition, our research examines how influential motivational and contextual factors intersect with other background characteristics of two-year college women to collectively shape their transfer intent. Our findings will inform the current knowledge base on the ways in which colleges and universities support and encourage upward mobility in STEM fields for two-year college women.

## Review of the literature and conceptual framework

In the following sections, we review relevant prior research that situates our study and informs the study's conceptual framework. Given the sparse literature directly addressing our research focus, we resort to the body of work dealing with choice of STEM fields as well as the small line of research on the general experiences of women in STEM fields at two-year colleges.

### Potential factors associated with two-year college women's STEM pursuits

Several factors emerge that hold strong theoretical promise to guide our inquiry. To begin, the choice to pursue STEM fields of study is influenced by students' motivational beliefs in math and science (Wang, 2013b). In particular, confidence in math and science has been shown to have a profound influence on students' decisions to pursue STEM fields in the first place (Moakler and Kim, 2014), especially for women and minorities (Hackett and Betz, 1989). Specifically for women in STEM fields at community colleges, another factor that may strongly shape their success is the contextual supports they receive and barriers they encounter, especially through their interactions with and exposure to a variety of institutional agents such as faculty, advisors, and student peers, along with family and friends. For example, as Packard et al. (2011) found in their qualitative research, two-year college female students in STEM fields mostly persist throughout the first year in STEM fields, due in large part to having supportive faculty, peers, and family members, as well as flexible work schedules, among other factors. In the context of our study that addresses the intent to transfer in STEM, it stands to reason that such contextual exposure focused around transfer, that is, transfer-oriented interactions (Laanan et al., 2010; Starobin et al., 2014), may represent a prominent factor at play.

### Transfer intent of women in STEM fields at two-year colleges

While previous research has demonstrated the importance of understanding factors that influence the educational intent among two-year college students (Wang, 2012, 2013a,b), we have limited knowledge that sheds light on the intent to transfer upward in STEM fields. This gap in the literature is remarkable, as two-year college students' educational goals and intent are diverse and fluid, and thus need to be understood and supported in a highly situated and purposeful way (Wang, 2015). Furthermore, considering the myriad challenges identified in prior research around women in STEM fields (e.g., Hoffman et al., 2010; Packard et al., 2011), it is pivotal to examine personal and contextual influences on intentions to transfer into STEM fields at four-year institutions among women enrolled in two-year colleges. These intentions are often disrupted by life experiences that shift the pathways and even alter the intentions of women in their academic and career pursuits (e.g., Wickersham and Wang, 2016). Unfortunately, to date, this is a sorely underdeveloped line of inquiry that warrants further exploration.

### Multiple roles and background characteristics of women in STEM at two-year colleges

Women beginning their STEM careers at two-year colleges tend to possess many forms of diversity, which may shape their transfer intent differently. For example, family obligations are common constraints that discourage women from enrolling in further education more often than men (Ferriman et al., 2009; Sax, 2012). In particular, two-year college students' marital status is negatively related to the likelihood of graduating or transferring, even when their postsecondary academic experience is accounted for (Chan and Wang, 2016). Also, childcare obligations have been identified as a critical factor for women's career plans in STEM fields (Sonnert, 1995). Though the flexible course schedule in two-year colleges would fit the needs of women who have children, they still struggle with securing childcare options because fewer than half of two-year colleges offer on-campus childcare (Costello, 2012; McKinney and Novak, 2013; Martin et al., 2014). In addition, many female students attending two-year colleges work long hours; as a result, women may feel like they have to compromise either their upward mobility or their ideal family lives in order to achieve some sort of life balance (Tajlili, 2014). Consequently, they may not be able to enroll full-time or contribute a reasonable amount of time and energy to academics due to these demands. The financial need would put female single parents in a particularly disadvantaged position, as they concurrently face multiple time and financial constraints. Finally, first-generation college women may not receive a reaffirming opinion from their parents about the benefits of a postsecondary credential, in that their parents are not able to be role models, or do not have the knowledge and experience of postsecondary education, to encourage their offspring to pursue a postsecondary degree in STEM (Shapiro and Sax, 2011).

Overall, our review of the literature indicates that the two-year college pathway to baccalaureate STEM studies for women has been understudied, and furthermore, research is scant that addresses how two-year college women's other characteristics may shape their transfer intent. To achieve a better and more accurate understanding of the educational intent and pathways of women in STEM fields, we must bring two-year colleges into the equation, as well as account for the many other backgrounds of women STEM students in two-year colleges. Given women's many roles and responsibilities, having a greater grasp of how their other characteristics may help or hinder their transfer intent will allow higher education policy makers and practitioners to better address women's needs accordingly. Informed by and contributing to the literature, our study will shed light on factors associated with the transfer intent among female two-year college students in STEM, as well as determine how their other backgrounds may intersect with learning and motivational factors that influence transfer intent. Our study thus adds to the literature by illuminating the potential barriers and supports facing two-year college women in STEM in their educational pursuits.

## Conceptual framework

**Figure 1:**
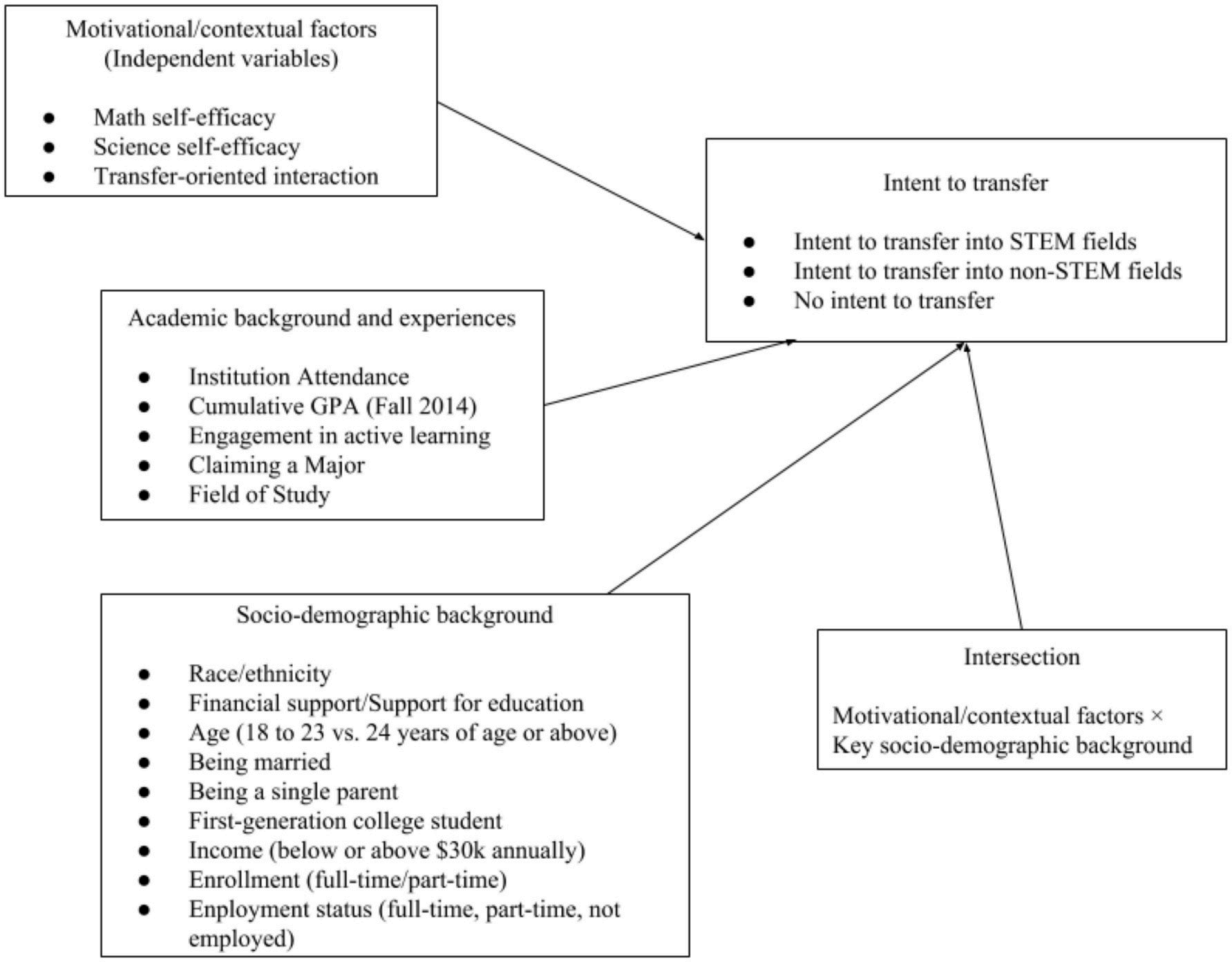
**Conceptual framework**.

Figure 1 represents the conceptual framework that guides our study, which is informed by the social cognitive career theory (SCCT; Bandura, 1986; Lent et al., 1994, 2000) that postulates the central role of cognitive and personal factors in the process of individuals' career development, as well as pertinent prior literature in higher education research. Several constructs in SCCT are relevant for our study. Specifically, self-efficacy—individuals' belief in their own ability to accomplish a given task—is regarded as a pivotal factor underlying individuals' academic and career development. This assumption has been supported by previous studies on academic aspirations among two-year college students entering STEM fields (e.g., Hagedorn and DuBray, 2010; MacPhee et al., 2013; Wang, 2013a,b). Given the specific focus of our study, we highlight female students' *self-efficacy beliefs in math and science*, as women's self-beliefs in their proficiency in these two areas have been found to undergird their aspiration to enter STEM fields (Blickenstaff, 2005; Wang, 2013b). Another key element of our conceptual framework is *transfer-oriented interaction*, indicating students' engagement in interactions with institutional agents (e.g., instructors, academic advisors), or efforts to gather transfer information or to prepare essential materials needed for transfer. Transfer-oriented interaction may demonstrate the actual efforts students spend on preparing themselves to successfully transfer, and is a critically important construct to inform research on student transfer to four-year institutions (Laanan, 2007; Starobin et al., 2014).

To account for other relevant factors that may influence two-year college students' intent to transfer, our conceptual framework also includes students' academic background and experiences, such as the type of two-year institutions students attend, their engagement with active learning experiences (Prince, 2004; Hagedorn et al., 2008), their first term grade point average (GPA; e.g., Bean and Kuh, 1984), and their programs of study (Wang, 2016).

A number of socio-demographic background characteristics deemed critical for students' academic choices and pathways, as identified in prior literature, are also addressed in our framework, including the support for one's pursuit of postsecondary education, enrollment intensity, and students' employment status (Lent et al., 1994, 2000; Wang, 2009, 2013a). Furthermore, we focus on four background characteristics that are particularly pertinent to female students' pursuit of postsecondary education: race/ethnicity, marital status, single parent status, and first-generation status. We argue that these four background characteristics represent important aspects of women's identities and play a significant role when students develop their future academic goals and make career decisions. While some studies have started to empirically examine how women with these backgrounds fare differently in their academics compared with their peers (e.g., Oh and Lewis, 2011; Ruiz, 2013), how these identities interact with the noted motivational and contextual factors to inform women's academic and career pathway in STEM largely remains unclear (Cohen et al., 2014; see Ferriman et al., 2009; Costello, 2012, for recent examples). Without an examination of the intersections between these identities and the key motivational and contextual factors that are pertinent to students' transfer intent, researchers and practitioners may gain a false understanding that the relationship among these factors represents two-year college women as a homogenous group.

## Methods

Guided by the conceptual framework and prior literature, we examine the following two research questions. First, what are the factors associated with the intent to transfer to a four-year institution among female students beginning in STEM programs or courses at two-year colleges? We are particularly interested in exploring the relationship between transfer intent and three key independent variables: math self-efficacy, science self-efficacy, and transfer-oriented interaction; and hypothesize that these three factors are positively related to two-year college women's transfer intent. Second, how are the relationships between the three noted key independent variables and transfer intent moderated by background characteristics of two-year college women such as their race/ethnicity, first-generation status, marital status, and single parent status? We hypothesize that these background factors moderate the relationships, but given the lack of prior research, we do not assume specific directionality of the findings, as we approach the second question in a highly exploratory fashion.

### Data and sample

We analyzed data collected from the baseline survey of a statewide longitudinal study of students enrolled in two-year colleges with a transfer mission located in a Midwestern state. These colleges include two comprehensive two-year colleges and the two-year campuses within the state university system. First-time students who were enrolled in STEM courses in Fall 2014 were invited to participate in the study. Approximately 3000 students were targeted (i.e., 1000 students from each comprehensive two-year college and another 1000 students from all two-year campuses of the state university system). For institutions where enrollment is small within certain racial/ethnic groups or specific STEM fields, the sample was selected using a stratified sampling design with two strata: race/ethnicity and STEM fields. As a result, the sampling weight, which is the inverse of the probability of selecting a student from the target population, was calculated and applied across analyses. Data collected from the survey were also matched with students' administrative and transcript records.

The survey consisted of slightly over 100 items and was designed to measure two-year college students' learning experiences, motivational beliefs, and contextual factors that could influence their upward transfer. The survey's content validity was established based on extensive literature review and input from national experts specialized in STEM education issues, two-year colleges, and survey design. Furthermore, a pilot study was conducted in Summer 2014, involving a group of nearly 100 two-year college students in the state, including both survey data collection and cognitive interviews. These procedures helped establish initial evidence regarding the survey's content, face, and process validity.

Students in our target sample received the survey in Fall 2014 via a letter containing the survey URL. Students were offered a $5 cash pre-incentive, and another $10 incentive upon completing the survey. After the initial contact, we emailed non-respondents 1 week later, and repeated the process the following week with a postal mailing. Three weeks after initially contacting potential participants, we sent students another email. Survey packets were mailed 30 days following initial contact, and as a final contact, non-respondents received a last email with the survey URL 5 weeks after initial contact. In total, 56.6% of the target sample members, or 1668 students, completed the survey.

For the purpose of our study, the analytic sample includes a total of 696 females out of the 1668 students who completed the baseline survey (41.7%). In the unweighted sample, a majority of the students is White (69.1%), followed by Hispanic (14.7%), Asian (7.0%), Black (5.5%), and other racial/ethnic backgrounds (e.g., Native American, multi-racial; 3.7%). Over a quarter of the participants (27.9%) were above 24 years of age. About a third of the participants were first-generation students (32.0%), 11.4% of them were married, and 7.6% self-identified as single parent (see Table 1 for a summary of the unweighted sample characteristics).

**Table 1:** **Summary of sample characteristics and descriptive statistics**.

**Variable**	**%**	***M*** **(*SD*)**	**Skewness**	**Kurtosis**
**DEPENDENT VARIABLE: INTENT TO TRANSFER**				
Intent to transfer into STEM fields	37.6			
Intent to transfer into non-STEM fields	37.8			
No intent to transfer	24.4			
**INDEPENDENT VARIABLES**				
Math self-efficacy		3.71 (0.91)	−0.60	0.29
Science self-efficacy		3.67 (0.88)	−0.47	0.05
Transfer-oriented interaction		2.34 (0.91)	0.29	−0.57
**CONTROL VARIABLES: ACADEMIC BACKGROUND AND EXPERIENCES**				
Institution attendance: comprehensive two-year institution	60.5			
Institution attendance: two-year campuses within the state university system	39.5			
Cumulative GPA (Fall 2014)		2.99 (0.99)	−1.35	1.53
Engagement in active learning		3.31 (0.69)	−0.14	0.19
Have claimed a major	54.4			
Have not claimed a major	45.6			
Field of study: biological, agricultural, or environmental life science	47.7			
Field of study: computer or mathematical sciences	23.0			
Field of study: engineering or engineering technologies	6.1			
Field of study: physical sciences	23.3			
**CONTROL VARIABLES: SOCIO-DEMOGRAPHIC**				
Black	5.5			
Hispanic	14.7			
Asian	7.0			
Other race/ethnicity	3.7			
White	69.1			
Financial support		2.78 (1.36)	0.11	−1.18
Support for education from family		4.03 (1.19)	−1.04	−0.01
Support for education from peers		3.79 (1.11)	−0.77	−0.03
Being 18– 23 years of age	72.1			
Being over 24 years of age	27.9			
Being married	11.4			
Not married	88.6			
Being a single parent	7.6			
Not a single parent	92.4			
First-generation college student	32.0			
Non-first-generation college student	68.0			
Low income (below $30 k annually)	37.2			
Not low income (above $30 k annually)	62.8			
Full-time student	64.7			
Part-time student	35.3			
Employed: full-time	20.7			
Employed: part-time	59.1			
Employed: not employed	20.2			

### Measures

#### Transfer intent

Students' intent to transfer to a four-year institution was measured using two multiple-choice survey items. The first item is a dichotomous item asking whether participants have the intention to transfer to a four-year university (1 = yes, 0 = no). If participants indicated intent to transfer, they were prompted to answer which of the five fields of study they would like to transfer into, where the first four alternatives are considered STEM-related fields (1 = biological, agricultural, or environmental life sciences, 2 = computer or mathematical sciences, 3 = engineering or engineering technologies, 4 = physical sciences including chemistry, physics, astronomy, etc., 5 = other major program of study). Based on responses to these two items, we derived a categorical outcome variable with three scenarios: intent to transfer into STEM fields, intent to transfer into non-STEM fields, and no intent to transfer.

#### Math and science self-efficacy

Participants responded to two five-item scales measuring their self-efficacy in math and science (e.g., how confident are you that you can do well on math exams), respectively, on a 5-point Likert scale (1 = not at all, 5 = extremely). The two scales share the same item stem but the subject matter varies between math and science. Both scales have high internal consistency (Cronbach α's = 0.96 for both scales). The scale scores were calculated by averaging the score of each item separately for math and science self-efficacy. Both scales were regarded as important motivational factors and as two of the three key independent variables in the study.

#### Transfer-oriented interaction

As one of the three key independent variables, transfer-oriented interaction is a seven-item scale that measures the frequency of utilizing campus resources or contacting institutional agents (e.g., instructors, academic advisors) through which the participants could gather information of transferring to a four-year institution (e.g., how often do you use the following service provided by your college or campus: advising for future transfer to a four-year college, either walk-in or online) on a 5-point Likert scale (1 = never, 5 = very often). The scale has high internal consistency (Cronbach α = 0.86), and the mean score of the seven items was used as the scale score in the analyses.

#### Academic background and experiences

Five variables were controlled for as participants' academic background and experiences. These included participants' institution of attendance (attending a comprehensive two-year institution or the two-year campuses within the state university system; the latter was the reference group), cumulative GPA as of Fall 2014 on a 4.00 scale, the average score of the scale on students' engagement in active learning, whether the participants have claimed a major, and a set of dichotomous variables denoting their field of study (i.e., biological, agricultural, or environmental life sciences; computer or mathematical sciences; engineering or engineering technologies; physical sciences, including chemistry, physics, astronomy, etc.; students in the physical sciences were the reference group).

#### Socio-demographic backgrounds

Ten types of socio-demographic background, dummy-coded variables were controlled for, including race/ethnicity (separated into Black, Hispanic, Asian, and other race/ethnicity, with White being the reference group), financial support for education, emotional support for education separately from family members and peers, being over 24 years of age when enrolled in Fall 2014, being married, being a single parent, being a first-generation student, having an annual household income lower than $30,000, being a part-time student (i.e., enrolled in <30 credits per year, according to the definition provided by the institutions included in our study), being employed full- or part-time (having no employment as the reference group).

### Missing data

Missing data were scarce in the current study. More than 96% of the study participants had complete data from their survey responses and administrative records, and merely a total of 0.2% of the data points were missing. Little's missing-completely-at-random test (Little, 1988), conducted in SPSS 22.0, indicated that the data were missing completely at random (χ^2^ = 31.42, *df* = 21, *p* = 0.07). While list-wise deletion would not seriously bias the data distribution, to retain all 696 female survey respondents we conducted multiple imputation using M*plus* 7.4. Ten imputed data sets were generated and the pooled results are reported in the present study.

### Analysis

Confirmatory factor analysis (CFA) was first conducted in M*plus* 7.4 to verify the psychometric property of the math self-efficacy, science self-efficacy, transfer-oriented interaction, and engagement in active learning scales. The analysis was performed with weighted least squares means and variance adjusted estimation (WLSMV) to accommodate the nature of Likert scales and sampling weights. A four-factor solution was estimated, since the items were developed to measure four latent psychosocial and behavioral dispositions: self-efficacy (separately in math and science), transfer-oriented interaction, and engagement in active learning, where each scale corresponds to one latent construct. For a complete list of the survey items measuring each of the four latent factors, see Appendix A in Supplementary Material.

Second, a multinomial logistic regression model was analyzed with a categorical outcome variable indicating three scenarios (i.e., no intent to transfer, intent to transfer into non-STEM fields, and intent to transfer into STEM fields). Thus, in the multinomial logistic regression context, two discrete logistic regression models—respectively, no intent to transfer as opposed to intent to transfer into STEM, and intent to transfer into non-STEM as opposed to intent to transfer into STEM—were estimated simultaneously. The three key independent variables (i.e., transfer-oriented interaction, self-efficacy in math, and self-efficacy in science), other academic and background variables, along with a total of 21 interaction terms between each of the main independent variables and four key background variables (i.e., race/ethnicity, being married, single parent status, and first-generation student status). This multinomial logistic regression model was analyzed with robust maximum likelihood estimation (MLR) to accommodate the sampling weight, and the skewness and kurtosis of the continuous independent variables and covariates.

**Figure 2:**
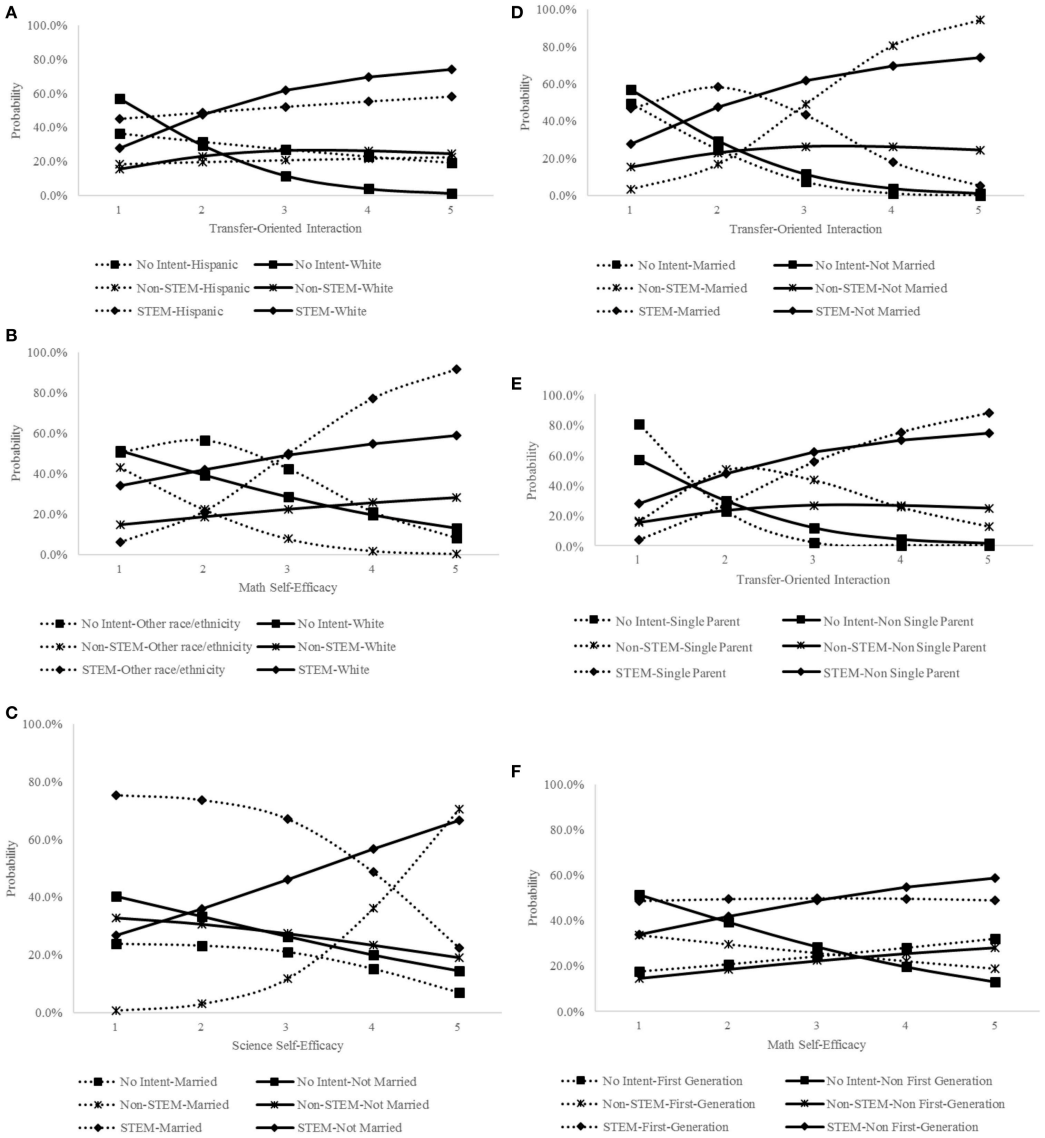
**Predicted probabilities of transfer intent based on the interaction effects between key independent variables and sociodemographic backgrounds. (A)** Interaction between transfer-oriented interaction and race/ethnicity. **(B)** Interaction between math self-efficacy and race/ethnicity. **(C)** Interaction between science self-efficacy and marital status. **(D)** Interaction between transfer-oriented interaction and marital status. **(E)** Interaction between transfer-oriented interaction and being single parent. **(F)** Interaction between math self-efficacy and first-generation status.

Odds ratio (OR) and projected changes in the probability of choosing a certain category in the outcome variable were calculated to further understand how the changes in a predictor were related to women's transfer intent. These statistics were reported for coefficients that achieved statistical significance at *p* < 0.10, *p* < 0.05, *p* < 0.01, and *p* < 0.001, specifically. Finally, for statistically significant interaction effects at the *p* < 0.05 level, we combined the two discrete logistic regression models, simultaneously estimated in our multinomial logistic regression analysis to plot the relative risk ratio and predicted probability (in Figure 2; and probability change in Appendix B in Supplementary Material) graphs of these interaction terms to describe the intersections between the key independent variables and the four background characteristics noted earlier. These estimates were derived based on Muthén and Muthén (1998–2015, pp. 495–499). To illustrate, the ORs of reporting *intent to transfer into non-STEM fields* and *no intent to transfer* at a given level of a key independent variable, when controlling for other variables, were calculated separately, both with *intent to transfer into STEM fields* as the reference category. Since there were three levels in the outcome variable, three ORs (two estimated and the reference category fixed at 1) were generated for a given level of the key independent variable. These ORs were then used to calculate the predicted probability of choosing a certain category of the outcome variable at a given level of the key independent variable, where each OR was the numerator and the summation of the three ORs the denominator, multiplied by 100%. Therefore, the summation of the three predicted probabilities is 100%. Given the involvement of all three ORs, conventionally, researchers plot all the predicted probabilities at a given level of the predictor regardless of its statistical significance. The process was repeated for different levels of the key independent variables and demographic background indicators.

### Limitations

Given the nature of our research design and data sources, readers should interpret our findings with the following limitations in mind. First, some context-specific factors may influence students' transfer intent, such as articulation agreements between two- and four-year institutions, especially in STEM fields. However, given the focus and scope of our study, we are not able to explicitly account for articulation agreements in the study. Related, while within our sample of two-year colleges with a transfer mission, we further took into consideration institutional differences by introducing a covariate distinguishing between comprehensive institutions vs. two-year campuses within the state university system, our findings do not necessarily hold for institutions where transfer is not an explicit part of their mission. Especially, our study sample may engage in transfer-oriented interactions in more homogeneous ways than students enrolled at two-year institutions without an explicit transfer mission, as students enrolled in those institutions may lack the same degree of transfer intent toward STEM or other fields given that these institutions' educational offerings and opportunities may divert students away from transfer as a major educational goal. Another limitation of our study is that transfer intent regarding STEM fields is measured in an aggregated fashion. While this approach allows us to retain analytical viability, factors related transfer intent across specific STEM fields, such as from science to engineering, are not explored. Finally, like with all observational studies, the results presented in this study are correlational in nature. Though some predictors may form a temporal relationship with the outcome variable, our analytical framework precludes causal interpretation.

## Results

### The factor structure of the four scales

The results from CFA revealed that the proposed four-factor solution fit the data well (χ^2^ = 1732.17, *df* = 458, *p* < 0.05; 90% CI of RMSEA = [0.06, 0.07], CFI = 0.98, TLI = 0.98), and the items were grouped together as designed. The standardized factor loadings ranged between 0.53 and 0.98, indicating that these items as a group are good measures of the latent construct. In short, these results demonstrated that the scales had acceptable divergent and construct validity.

### Results from the multinomial logistic regression

Largely aligned with our hypotheses, the results from the multinomial logistic regression demonstrated that the three motivational and contextual factors were significant and positive predictors for women's transfer intent (see Table 2 for a summary of the multinomial logistic regression). Further, this relationship seemed to be moderated by students' background characteristics (see Figure 2 and Appendix B in Supplementary Material for the graphs of the moderation effects). In essence, the relationship of transfer-oriented interaction and math self-efficacy to women's transfer intent differed between White and racial/ethnic minority students. For example, despite the positive relationship between transfer-oriented interaction and the intention to transfer into STEM fields, Black women were less likely to have intent to transfer into STEM fields than White students until Black students reported a moderate level of transfer-oriented interaction (at *p* < 0.10 level)^1^. Conversely, Hispanic students appeared to be more likely to report intent to transfer into STEM fields than their White peers, even when Hispanic students reported a relatively low level of engagement in transfer-oriented interaction (see Figure 2A). For students identified as other race/ethnicity (e.g., Pacific Islanders, Native Americans), they were more likely to report intent to transfer into STEM fields than their White peers at a medium to high level of math self-efficacy (see Figure 2B).

**Table 2:** **Summary of multinomial logistic regression**.

**Variables**	**STEM vs. no intent**			**STEM vs. non-stem**		
	***B*** **(*SE*)**	**OR**	**Prob. change (%)**	***B*** **(*SE*)**	**OR**	**Prob. change (%)**
**MOTIVATIONAL/CONTEXTUAL FACTORS**						
Math self-efficacy (Math SE)	0.48 (0.22)^*^	1.62	2.9	−0.03 (0.19)		
Science self-efficacy (Sci SE)	0.49 (0.25)^+^	1.63	9.0	0.36 (0.22)^+^	1.44	
Transfer-oriented interaction (TI)	1.19 (0.27)^***^	3.30	5.5	0.13 (0.18)		
**ACADEMIC BACKGROUND AND EXPERIENCES**						
Institution attendance: comprehensive two-year institution	−1.54 (0.37)^***^	0.22	−21.1	−0.04 (0.29)		
Cumulative GPA (Fall 2014)	0.13 (0.14)			−0.04 (0.14)		
Engagement in active learning	−0.01 (0.21)			0.41 (0.19)^*^	1.51	8.2
Have claimed a major	1.67 (0.29)^***^	5.34	39.6	2.76 (0.27)^***^	15.86	56.3
Field of study: biological, agricultural, or environmental life science	0.83 (0.34)^*^	2.30	14.0	0.59 (0.31)^+^	1.81	11.3
Field of study: computer or mathematical science	−0.28 (0.43)			−0.70 (0.40)^+^	0.50	−16.7
Field of study: engineering or engineering technologies	−0.79 (0.60)			1.14 (0.67)^+^	3.12	18.8
**SOCIO-DEMOGRAPHIC BACKGROUNDS**						
Black	−14.75 (6.62)^*^	0.00	5.4	−9.02 (4.88)^+^	0.00	−22.5
Hispanic	2.73 (2.20)			0.69 (1.99)		
Asian	−0.53 (3.17)			−3.58 (2.88)		
Other race/ethnicity	0.66 (3.66)			−3.14 (4.02)		
Financial support	−0.17 (0.10)^+^	0.85	−3.6	−0.13 (0.09)		
Support for education from family	−0.24 (0.14)^+^	0.79	−5.2	−0.13 (0.13)		
Support for education from peers	−0.16 (0.14)			−0.27 (0.14)^+^	0.76	−6.2
Being over 24 years of age	−0.75 (0.38)^+^	0.47	−17.3	0.57 (0.42)		
Being married	3.07 (1.90)			8.70 (3.32)^**^		0.1
Being a single parent	−1.85 (4.11)			−2.73 (4.20)		
First-generation college student	0.49 (1.68)			−2.43 (1.66)		
Low income (below $30 k annually)	0.22 (0.30)			0.92 (0.30)^**^	2.50	16.0
Part-time student	−0.22 (0.33)			−0.15 (0.32)		
Employed: full-time	−0.77 (0.41)^+^	0.46	−17.9	−0.59 (0.39)		
Employed: part-time	−0.15 (0.34)			0.13 (0.29)		
**INTERACTION TERMS**						
TI × Black	1.95 (1.10)^+^	7.03	–	1.04 (0.59)^+^	2.82	–
TI × Hispanic	−0.97 (0.42)^*^	0.38	–	−0.11 (0.35)		
TI × Asian	−0.89 (0.71)			0.12 (0.49)		
TI × Other race/ethnicity	−1.00 (0.90)			−1.41 (0.91)		
Math SE × Black	2.29 (1.17)^+^	9.90	–	1.41 (0.91)		
Math SE × Hispanic	−0.37 (0.39)			0.23 (0.37)		
Math SE × Asian	−0.03 (0.67)			0.35 (0.54)		
Math SE × Other race/ethnicity	0.65 (0.60)			1.93 (0.78)^*^	6.89	–
Sci SE × Black	0.77 (0.90)			0.12 (0.83)		
Sci SE × Hispanic	0.15 (0.49)			−0.31 (0.46)		
Sci SE × Asian	0.70 (0.83)			0.44 (0.74)		
Sci SE × Other race/ethnicity	−0.18 (0.61)			0.47 (1.15)		
TI × Married	−0.29 (0.45)			−1.49 (0.55)^**^	0.23	–
TI × Single parent	2.06 (0.80)^*^	7.82	–	0.72 (0.62)		
TI × First-generation	0.22 (0.37)			0.38 (0.31)		
Math SE × Married	−0.10 (0.53)			0.39 (0.66)		
Math SE × Single parent	−0.76 (0.48)			−0.22 (0.52)		
Math SE × First-generation	−0.63 (0.32)^*^	0.53	–	0.17 (0.30)		
Sci SE × Married	−0.48 (0.54)			−1.81 (0.82)^*^	0.16	–
Sci SE × Single parent	0.08 (0.94)			0.21 (0.98)		
Sci SE × First-generation	0.29 (0.41)			0.24 (0.42)		
(Intercept)	−3.77 (1.35)			−2.97 (1.15)		

*OR, odds ratio. Probability change of statistically significant variables was calculated by comparing with the other group (for binary variables) or contrasting students scored at mean against who scored 1 point above the mean (for continuous variables). However, because of the interaction effects, some directions of the probability change of main effects are not equal to that of the corresponding coefficients. Probability change of the interaction terms was not calculated*.

+ *p < 0.10*,

* *p < 0.05*,

** *p < 0.01*,

*** *p < 0.001*.

The relationship of science self-efficacy and transfer-oriented interaction to transfer intent also seems to vary based on marital status. Married women appeared to be less likely to report intent to transfer into STEM fields than those who are not married, despite the positive linkage of science self-efficacy and transfer-oriented interaction to transfer intent (see Figures 2C,D). Female students who were also single parents, compared with those who were not, seemed to be more likely to report intent to transfer into STEM fields if they report a relatively high level of transfer-oriented interaction (see Figure 2E). Finally, though first-generation female students would be more likely to report intent to transfer into STEM fields than non-STEM ones, this probability would lag behind that of non-first-generation students when they both scored relatively high in math self-efficacy (see Figure 2F).

## Discussion

In regard to our first research question on the relationship between the three key contextual and motivational factors and transfer intent, our findings align with prior research that transfer-oriented interaction aids in the process leading up to transfer (e.g., Kruse et al., 2015). Our results also show that math self-efficacy and science self-efficacy may enhance intent to transfer into STEM fields, which also resonates with existing empirical evidence pointing to the foundational role of self-beliefs in math and science performance in shaping students' STEM educational trajectories and outcomes (e.g., Peters, 2013; Wang, 2013a,b; Starobin et al., 2014; Larson et al., 2015; Lent et al., 2015). While these general patterns are what we would expect, it is important to note that, overall, these three factors seem to exert very similar positive influence on the intent to transfer into STEM as well as transfer into other fields. That is, transfer-oriented interaction and self-efficacy beliefs help distinguish between intent to transfer in general and having no intent to transfer, but not among major fields of study into which students intend to transfer. This is an intriguing finding, considering the fact that the women in our study were all exposed to STEM courses and/or programs. While the increased amount of transfer-oriented interaction or self-efficacy beliefs does seem to boost transfer intent in general, it does not necessarily push women further toward the STEM transfer path. This result suggests that other factors may matter more for transfer-aspiring two-year college women as they decide which area they want to pursue at the four-year level, such as academic interests and concerns about future careers, which have been identified as contributing factors to college major choice (Freeman and Hirsch, 2008; Martinez, 2012; Wang, 2013b). Considerations around these aspects may outweigh transfer-oriented interaction and self-efficacy beliefs as two-year college women map out their future academic plans.

With our second research question, we explored the potential ways in which two-year college women's other identity backgrounds (i.e., racial/ethnic background, marital status, single parent status, and first-generation status) may moderate the relationships between intent to transfer and the three contextual and motivational factors. As reported earlier, we uncovered some potential racial/ethnic differences regarding how transfer-oriented interaction and math self-efficacy may shape intent to transfer. An important takeaway is that, while transfer-oriented interaction is an important predictor of transfer intentions in general, it is a critically important type of interaction for Black women at two-year colleges. On one hand, the lack of such interaction is especially detrimental to the baccalaureate STEM aspirations among Black women who already feel isolated by being female and minority in White and male-dominated STEM programs (Jackson et al., 2013). On the other hand, based on our study, when students engage in transfer-oriented interaction to a great degree, it may serve as an amplifier that has a substantial boosting effect to STEM transfer intent of Black women. This complex dynamic also holds for how math self-efficacy is related to transfer intent among Black women relative to their White counterparts, and reinforces the promise and power of continued efforts to enhance academic confidence for women in order to increase the representation of women in STEM fields.

As for married women, an increasing level of transfer-oriented interaction appears to boost their intent to transfer into non-STEM fields, as opposed into STEM. In this sense, while transfer-oriented interaction still cultivates the intent to pursue a four-year, as women increasingly interact with institutional agents to talk about transfer, it serves to direct women into different transfer pathways depending on their marital status. Also interesting to note is that an increased level of science self-efficacy operates in the same way as transfer-oriented interaction based on marital status. This counterintuitive finding may be explained by the widely-held perceptions of scientists and engineers working long hours (Terosky et al., 2014). It is possible that, as women learn about the expectations of the field, despite their growing confidence in career competence—as evidenced in their science self-efficacy—they may stay away from a STEM career for fear of not being able to balance life and work (Xie and Shauman, 2003).

Single parent status also emerged to be a noteworthy moderator of the relationship between transfer-oriented interaction and intent to transfer. The surge in probability of single parents reporting intent to transfer into STEM with greater levels of transfer-oriented interaction demonstrates the importance of contextual support in preparing female single parents for having a future in STEM careers, especially given that single parents have to juggle multiple obligations (Creamer and Laughlin, 2005; Hagedorn and Purnamasari, 2012).

Finally, with regard to first-generation status, our results indicate that math self-efficacy is more relevant to non-first-generation students' transfer intention, whereas its influence on first-generation women is rather minimal. This finding is interesting, considering that self-efficacy has been long established as a reliable and important precursor to students' academic aspirations and achievement (e.g., Hackett, 1985; Lent et al., 2015), especially in STEM fields (Heinze and Hu, 2009; Larson et al., 2015; Sax et al., 2016). It is plausible that first-generation two-year college women are more inclined to pursue STEM fields for reasons beyond their self-perceptions of their math abilities. Alternatively, given that our study focuses on transfer intent early in these women's two-year college careers, it is also likely that the relevance of math self-efficacy will gain more ground as these students spend more time navigating through the STEM curriculum.

Taken together, our findings suggest that, by and large, the three motivational and contextual factors of our key interest, transfer-oriented interaction, self-efficacy in math, and self-efficacy in science, are good predictors of two-year college women's intent to transfer into STEM fields. In addition, there are more nuanced findings that pertain to these three key factors when we take into consideration of women's other identities. In particular, while self-efficacy beliefs demonstrate solid predictive power for female students' intent to transfer in general, the role they play varies in magnitude across student subgroups. As a core component of the social cognitive theory, self-efficacy has been widely applied in research examining academic and career choices. Our study validates self-efficacy's established utility, but more importantly, it illuminates the imperative need to adopt a more nuanced approach to examining self-efficacy among diverse groups of community college women along the STEM transfer pathway. Further, factors other than self-efficacy, such as women's identity as a STEM learner and sense of belonging within STEM fields, need to be further explored in future inquiry.

## Implications for policy and practice

### Engaging students in transfer-oriented interaction to fuel transfer intent

In light of the substantial role transfer-oriented interaction plays in shaping women's transfer intent in our study, it is critical to identify ways to engage students in interaction with important institutional agents regarding transfer among women enrolled in STEM programs at two-year institutions. Accordingly, it is important that two-year colleges offer resources to help women increase their knowledge about transfer into four-year STEM programs, through intentional and proactive academic and transfer advising that helps illuminate viable transfer pathways (Packard et al., 2012; Packard and Jeffers, 2013). In her qualitative study, Jackson (2013) found that interpersonal exchange, such as consistent communication and information sharing within the community, as opposed to solitary activities (e.g., looking up information online), is the major venue through which Black two-year college women prepare for upward transfer and gain career knowledge in STEM fields. We would further recommend that in this endeavor, faculty, and transfer advisors work together to create a network of support, bridging the classroom with transfer advising to chart a seamless information network for students. Stronger collaboration between faculty and transfer advisors will allow students to receive holistic and consistent support and information, as two-year college women do not have extended time to spend on campus and navigate a multitude of functional areas.

For female STEM students initially with a low level of transfer-oriented interaction, a personalized transfer plan based on students' skills and interests may serve as a tangible way to build an understanding of upward transfer. Also, we advise two-year colleges to identify female role models in students who negotiated the transfer process and are completing baccalaureate STEM degrees. Given our findings, this practice may be especially valuable for women from backgrounds that are historically perceived as “disadvantaged” in STEM fields, such as being married. Engaging with peer role models sharing similar identity backgrounds who transferred into STEM fields may help dismiss notions that they are not capable or not able to attain support, should they pursue a baccalaureate STEM pathway.

### Cultivating math and science self-efficacy

For women in general, and women of color in particular, math and science self-efficacy beliefs play a significant and positive role in shaping intent to transfer into STEM fields. Therefore, more intensive efforts are called for to provide women pursuing STEM fields with ample opportunities to develop academic confidence. Arguably, faculty will serve as the key change agents in this process, given that the beliefs, practices, and attitudes of faculty have the power to diminish or strengthen self-efficacy for math and science among women of color (Bensimon, 2005). Moreover, STEM instructors' encouragement was deemed more valuable to students than that from their relatives and friends (Jenson et al., 2011).

Programs that immerse women in practical and applied experiences may also aid in this process. For example, undergraduate research experience may offer pivotal mastery experience that underscores self-efficacy (Bandura, 1986; Chemers et al., 2011). More intentional programming options that align female two-year college students with female instructors and scientists for research-related internships could provide women with mentors and role models they need to envision themselves successfully pursuing STEM fields. In a similar vein, if two-year colleges can pair female students with influential figures who share similar racial/ethnic, parental, or marital backgrounds, students may thus gain invaluable vicarious experiences (Bandura, 1986), which will increase their self-efficacy.

Vicarious learning experiences also stem from participating on teams and collaborating with peers (Jenson et al., 2011). In the present study, we also found that one's engagement in active learning is related to a higher probability of having intent to transfer into STEM fields as opposed to non-STEM fields. Peers provide support, learn from one another, and feel more confident in their abilities when they saw classmates succeed (Jenson et al., 2011). This suggests that, for women in STEM programs at two-year colleges, interactive curricular approaches adopted by STEM faculty may be beneficial in increasing both transfer intent and self-efficacy in math and science. However, faculty should be cautioned to allow students to designate their own teams, so women do not feel isolated or rejected if placed in groups of otherwise all male students.

## Implications for future research

Being one of the first empirical efforts disentangling two-year college women's transfer intent and how its associated factors intersect with women's other identity backgrounds, our study both sheds preliminary light on this understudied topic and illuminates two major future areas of inquiry that can build upon our findings.

First and foremost, more research is needed to further tease out how two-year college women's multiple roles and background characteristics shape their educational aspirations and eventual outcomes along the STEM pathway. Two-year college women are not a homogenous group, and as we continue to wrestle with the best approaches to closing the gender gap within STEM fields, researchers and policymakers alike must be cognizant of, and further examine, the diversity that exists within this subpopulation in order to tackle the unique challenges they might face based on their distinctive backgrounds. For example, our study shows that, while in general transfer-oriented interaction exerts a positive connection with intent to transfer, the extent to which it makes a difference varies based on women's other identity backgrounds. In light of our findings, more in-depth empirical effort should be devoted to studying the kinds of interventions that effectively help engage Black women and single parent women in transfer-oriented interaction; whereas the type of research that serves to better understand married women's transfer intent and pathways needs to extend beyond their transfer-oriented interaction and into women's perceptions of the demands and rewards of a STEM career, and how these factor into their pursuit of a STEM educational pathway. In sum, much remains to be researched in regard to the ways in which transfer intent among women in the STEM pipeline is shaped in connection with their other identities and roles.

In addition and related to the first point, more research needs to be conducted on the kinds of supports that are helpful for women in general and parents in particular to alleviate their hesitance in pursuing a STEM career. As our results show, single parent women indicate stronger transfer intent into STEM fields when their engagement in transfer-oriented interaction is frequent. On the other hand, married women have a low probability of reporting intent to transfer into STEM fields, despite a high level of engagement in transfer-oriented interaction. This finding may be attributed to a lack of recognition among the professional STEM community of the dual role of women balancing familial and work responsibilities (Xu, 2015), which may direct women to other fields of study as they become more informed about STEM career demands. Future studies may explore this dichotomy, as well as how two-year colleges can work with employers in STEM fields in creating empowering and welcoming environments, coupled with necessary educational and career resources that inform and encourage female students as they navigate their educational pathways into a STEM career.

## Conclusion

Despite the two-year college promise in solving the gender gap in baccalaureate STEM attainment through the upward transfer function, empirical research is sorely lacking on what influences the transfer intent among women beginning in STEM at two-year colleges. Our study sets out to fill this void in the research literature and further unravel the role women's other backgrounds play in the development of their transfer aspirations. The findings from this study indicate that women who are highly engaged with transfer-oriented interaction were more likely to report intent to transfer into STEM fields, making it especially critical that institutions provide transparent pathways for students to access resources and information about the transfer process. The findings also indicate that the relationship between math self-efficacy and intent to transfer into STEM fields is positive and significant. Women's self-beliefs in their proficiency in math may indeed shape their intent to continue in fields that require math courses at a higher level. Given that self-efficacy in math is shaped over time and through academic experiences, it is imperative that two-year colleges provide opportunities for students to be successful in foundational math and science courses. In addition, more nuanced findings emerge from our study when we take into consideration of women's other identities. For example, the relationship of math self-efficacy and transfer-oriented interaction to intent to transfer into STEM fields varied based on racial/ethnic backgrounds. These differences, as we discussed earlier, hold important implications for the ways in which researchers, policymakers, and practitioners study and inform students about pathways to STEM transfer.

As two-year colleges become more recognized as a pathway to four-year degrees in STEM fields, concentrated efforts are called for to support women who are traditionally underrepresented in STEM fields through the transfer pipeline. Future research is needed to dissect trends in the transfer decision process for women enrolled in two-year colleges, taking into account their many identities. More specifically, two-year colleges need to examine more deeply how women's identities interact with motivational and contextual factors that contribute to transfer ambitions, so as to better support women in their knowledge about upward transfer into STEM fields.

## Ethics statement

This study was carried out in accordance with the recommendations of UW-Madison's Institutional Review Board with written informed consent from all subjects. All subjects gave written informed consent in accordance with the Declaration of Helsinki. The protocol was approved by UW-Madison's Institutional Review Board.

## Author contributions

XW was primarily responsible for conceiving the study as well as collecting and analyzing the data. HSC contributed to the study design and conducted data analysis. SJS and BRN contributed to interpretation of the data. All authors wrote and revised the manuscript.

## Funding

This study is funded by the University of Wisconsin-Madison Graduate School Alumni Research Foundation and the National Science Foundation, grant No. DUE-1430642.

### Conflict of interest statement

The authors declare that the research was conducted in the absence of any commercial or financial relationships that could be construed as a potential conflict of interest.

## Footnotes

^1^We acknowledge that, based on the conventions of statistical reporting in psychology, the commonly adopted threshold for reaching statistical significance is 0.05. However, given the relatively small subsample sizes (such as that of Black women) and the exploratory nature of our study, we chose to also report parameter estimates with a *p*-value smaller than 0.10, in order to capture potentially meaningful relationships that can be further explored in future inquiry.
