# Supplementary material for: A Nuanced Look at Women in STEM Fields at Two-Year Colleges: Factors That Shape Female Students' Transfer Intent
Source: Front Psychol. 2017 Feb 6;8:146. doi: 10.3389/fpsyg.2017.00146 (PMC5292428; doi:10.3389/fpsyg.2017.00146)
Supplement: Supplementary file 1 [file DataSheet1.docx]

**Appendix A**

Survey Items Underlying Latent Factors in the Study

| Survey Items Corresponding to Factors | Loading |
| --- | --- |
| ***Math self-efficacy* (α = .96)**  How confident are you that you… |  |
| … have the ability to master the material taught in math? | .92 |
| … can do well on math exams? | .95 |
| … can complete math assignments successfully? | .91 |
| … can receive a good grade in college math courses? | .96 |
| … can perform well in course activities in math classes? | .97 |
| ***Science self-efficacy* (α = .96)**  How confident are you that you… |  |
| … have the ability to master the material taught in science? | .92 |
| … can do well on science exams? | .94 |
| … can complete science assignments successfully? | .92 |
| … can receive a good grade in college science courses? | .98 |
| … can perform well in course activities in science classes? | .95 |
| ***Transfer-oriented interaction* (α = .86)**  How often do you… |  |
| … use advising for future transfer to a four-year college, either walk-in or online | .83 |
| … use published transfer information or guidelines | .83 |
| … use transfer credit assistance, which helps you in determining how your course credits transfer to other colleges and universities | .85 |
| … contact each of the following individuals to discuss matters related to transfer to a four-year college or university? Instructors | .74 |
| … contact each of the following individuals to discuss matters related to transfer to a four-year college or university? Student peers | .66 |
| … contact each of the following individuals to discuss matters related to transfer to a four-year college or university? Academic advisors or counselors | .77 |
| … contact each of the following individuals to discuss matters related to transfer to a four-year college or university? Family members or friends | .59 |
| ***Engagement in active learning* (α= .90)**  How often do courses require you to… |  |
| … apply what you have learned to real-life situations? | .61 |
| … present what you have learned to instructor and peers? | .64 |
| … explore key concepts, data, beliefs, or values within small groups? | .70 |
| … think about instructors question on your own first, and then discuss the question with peers before the instructor explains the answer to the class? | .72 |
| … identify what you already know, what you need to know, and how and where to access new information in order to solve a given problem? | .63 |
| … consider, compare, and generate multiple potential solutions to a given problem? | .73 |
| … integrate skills and knowledge learned to solve problems? | .69 |
| … work in groups to research necessary background material in order to solve complex, realistic problems? | .72 |
| … draw diagrams to visually show the connection between a new concept and other concepts that you already learned? | .53 |
| … gather information from a variety of sources? | .69 |
| … draw conclusions and make decisions given a detailed description of a situation? | .73 |
| … evaluate student peers written work? | .53 |
| … work on real-world problems? | .65 |
| … work on your own projects or experiments? | .59 |
| … choose your own topics or projects to investigate? | .63 |
| *Note*. Factor loadings are standardized. Model fit indices: χ^2^ = 1732.17, *df* = 458, *p* < .001; RMSEA = .06 (90% CI = [.06, .07]); CFI = .98; TLI = .98 | |

**Appendix B**

Probability Change of Interaction Effects

(A) Interaction between transfer-oriented interaction and race/ethnicity

(B) Interaction between math self-efficacy and race/ethnicity

(C) Interaction between science self-efficacy and marital status

(D) Interaction between transfer-oriented interaction and marital status

(E) Interaction between transfer-oriented interaction and being single parent

(F) Interaction between math self-efficacy and first-generation status

*Note*. A positive probability change indicates a heightened trend of predicted probability of reporting a certain transfer intent, and vice versa.
